# Supplementary material for: The sports nutrition knowledge of large language model (LLM) artificial intelligence (AI) chatbots: An assessment of accuracy, completeness, clarity, quality of evidence, and test-retest reliability
Source: PLoS One. 2025 Jun 13;20(6):e0325982. doi: 10.1371/journal.pone.0325982 (PMC12165421; doi:10.1371/journal.pone.0325982)
Supplement: S1 File — Supplemental Tables for Statistical Analysis. S1 Table. The ANOVA summary table for Experiment 1. S2 Table. The partial eta-squared values for the ANOVA model for Experiment 1. S3 Table. Pairwise comparisons (inc. Cohen’s d effect sizes) for ChatbotID in the ANOVA model for Experiment 1. S4 Table. Pairwise comparisons (inc. Cohen’s d effect sizes) for TestDay in the ANOVA model for Experiment 1. S5 Table. Pairwise comparisons (inc. Cohen’s d effect sizes) for Domain in the ANOVA model for Experiment 1. S6 Table. Pairwise comparisons (inc. Cohen’s d effect sizes) for PromptType in the ANOVA model for Experiment 1. S7 Table. Pairwise comparisons (inc. Cohen’s d effect sizes) for the ChatbotID * Prompt_type interaction in the ANOVA model for Experiment 1. S8 Table. The LMM model summary table for Experiment 2. S9 Table. Pairwise comparisons for ChatbotID in the LMM model for Experiment 2. S10 Table. Pairwise comparisons for ExamDomain in the LMM model for Experiment 2. S11 Table. Pairwise comparisons for TestDay in the LMM model for Experiment 2. S12 Table. Effect sizes (r) and power for ChatbotID in the LMM model for Experiment 2. S13 Table. Effect sizes (r) and power for ExamDomain in the LMM model for Experiment 2. S14 Table. Effect sizes (r) and power for TestDay in the LMM model for Experiment 2. (DOCX) [file pone.0325982.s001.docx]

# Supplemental Tables

## Supplemental Table S1 – The ANOVA summary table for Experiment 1.

ANOVA model

Df Sum Sq Mean Sq F value Pr(>F)

ChatbotID 5 13.30 2.6606 7.659 6.52e-07 ***

TestDay 1 0.00 0.0038 0.011 0.9169

Domain 1 0.06 0.0629 0.181 0.6707

Prompt_type 1 0.04 0.0437 0.126 0.7230

ChatbotID:TestDay 5 0.52 0.1043 0.300 0.9127

ChatbotID:Domain 5 0.87 0.1737 0.500 0.7763

TestDay:Domain 1 0.02 0.0215 0.062 0.8035

ChatbotID:Prompt_type 5 3.64 0.7275 2.094 0.0651 .

TestDay:Prompt_type 1 0.03 0.0303 0.087 0.7680

Domain:Prompt_type 1 0.58 0.5788 1.666 0.1975

ChatbotID:TestDay:Domain 5 0.25 0.0492 0.142 0.9824

ChatbotID:TestDay:Prompt_type 5 0.04 0.0078 0.022 0.9998

ChatbotID:Domain:Prompt_type 5 2.67 0.5344 1.538 0.1766

TestDay:Domain:Prompt_type 1 0.16 0.1578 0.454 0.5007

ChatbotID:TestDay:Domain:Prompt_type 5 0.69 0.1390 0.400 0.8488

Residuals 432 150.08 0.3474

---

Signif. codes: 0 ‘***’ 0.001 ‘**’ 0.01 ‘*’ 0.05 ‘.’ 0.1 ‘ ’ 1

## Supplemental Table S2 – The partial eta-squared values for the ANOVA model for Experiment 1.

Partial eta-squared

# Effect Size for ANOVA (Type I)

Parameter | Eta2 (partial) | 95% CI

--------------------------------------------------------------------

ChatbotID | 0.08 | [0.04, 1.00]

TestDay | 2.52e-05 | [0.00, 1.00]

Domain | 4.19e-04 | [0.00, 1.00]

Prompt_type | 2.91e-04 | [0.00, 1.00]

ChatbotID:TestDay | 3.46e-03 | [0.00, 1.00]

ChatbotID:Domain | 5.75e-03 | [0.00, 1.00]

TestDay:Domain | 1.43e-04 | [0.00, 1.00]

ChatbotID:Prompt_type | 0.02 | [0.00, 1.00]

TestDay:Prompt_type | 2.02e-04 | [0.00, 1.00]

Domain:Prompt_type | 3.84e-03 | [0.00, 1.00]

ChatbotID:TestDay:Domain | 1.64e-03 | [0.00, 1.00]

ChatbotID:TestDay:Prompt_type | 2.59e-04 | [0.00, 1.00]

ChatbotID:Domain:Prompt_type | 0.02 | [0.00, 1.00]

TestDay:Domain:Prompt_type | 1.05e-03 | [0.00, 1.00]

ChatbotID:TestDay:Domain:Prompt_type | 4.61e-03 | [0.00, 1.00]

- One-sided CIs: upper bound fixed at [1.00].

## Supplemental Table S3 – Pairwise comparisons (inc. Cohen’s d effect sizes) for ChatbotID in the ANOVA model for Experiment 1.

Pairwise comparisons for ChatbotID

$emmeans

ChatbotID emmean SE df lower.CL upper.CL

ChatbotA 1.068 0.0662 432 0.938 1.198

ChatbotB 1.223 0.0662 432 1.093 1.354

ChatbotC 0.754 0.0662 432 0.624 0.884

ChatbotD 1.078 0.0662 432 0.948 1.208

ChatbotE 1.197 0.0662 432 1.067 1.327

ChatbotF 0.901 0.0662 432 0.771 1.032

Results are averaged over the levels of: TestDay, Domain, Prompt_type

Confidence level used: 0.95

$contrasts

contrast estimate SE df t.ratio p.value

ChatbotA - ChatbotB -0.15569 0.0937 432 -1.662 0.5576

ChatbotA - ChatbotC 0.31365 0.0937 432 3.349 0.0113

ChatbotA - ChatbotD -0.00999 0.0937 432 -0.107 1.0000

ChatbotA - ChatbotE -0.12945 0.0937 432 -1.382 0.7378

ChatbotA - ChatbotF 0.16633 0.0937 432 1.776 0.4825

ChatbotB - ChatbotC 0.46934 0.0937 432 5.011 <.0001

ChatbotB - ChatbotD 0.14570 0.0937 432 1.556 0.6283

ChatbotB - ChatbotE 0.02624 0.0937 432 0.280 0.9998

ChatbotB - ChatbotF 0.32203 0.0937 432 3.438 0.0084

ChatbotC - ChatbotD -0.32364 0.0937 432 -3.455 0.0079

ChatbotC - ChatbotE -0.44310 0.0937 432 -4.731 <.0001

ChatbotC - ChatbotF -0.14731 0.0937 432 -1.573 0.6170

ChatbotD - ChatbotE -0.11946 0.0937 432 -1.275 0.7983

ChatbotD - ChatbotF 0.17632 0.0937 432 1.883 0.4143

ChatbotE - ChatbotF 0.29579 0.0937 432 3.158 0.0209

Results are averaged over the levels of: TestDay, Domain, Prompt_type

P value adjustment: tukey method for comparing a family of 6 estimates

Since 'object' is a list, we are using the contrasts already present.

Cohen's d effect sizes for ChatbotID comparisons

contrast effect.size SE df lower.CL upper.CL

(ChatbotA - ChatbotB) -0.2641 0.159 432 -0.5770 0.0487

(ChatbotA - ChatbotC) 0.5321 0.160 432 0.2178 0.8465

(ChatbotA - ChatbotD) -0.0169 0.159 432 -0.3293 0.2954

(ChatbotA - ChatbotE) -0.2196 0.159 432 -0.5323 0.0930

(ChatbotA - ChatbotF) 0.2822 0.159 432 -0.0307 0.5951

(ChatbotB - ChatbotC) 0.7963 0.161 432 0.4794 1.1131

(ChatbotB - ChatbotD) 0.2472 0.159 432 -0.0656 0.5600

(ChatbotB - ChatbotE) 0.0445 0.159 432 -0.2678 0.3569

(ChatbotB - ChatbotF) 0.5464 0.160 432 0.2319 0.8608

(ChatbotC - ChatbotD) -0.5491 0.160 432 -0.8636 -0.2346

(ChatbotC - ChatbotE) -0.7518 0.161 432 -1.0681 -0.4354

(ChatbotC - ChatbotF) -0.2499 0.159 432 -0.5627 0.0628

(ChatbotD - ChatbotE) -0.2027 0.159 432 -0.5153 0.1099

(ChatbotD - ChatbotF) 0.2992 0.159 432 -0.0138 0.6121

(ChatbotE - ChatbotF) 0.5018 0.160 432 0.1877 0.8160

Results are averaged over the levels of: TestDay, Domain, Prompt_type

sigma used for effect sizes: 0.5894

Confidence level used: 0.95

Cohen's d & Power for ChatbotID comparisons (alpha = 0.05)

contrast effect_size SE df power

1 ChatbotA - ChatbotB -0.2641 0.159 432 0.12871389

2 ChatbotA - ChatbotC 0.5321 0.160 432 0.37471290

3 ChatbotA - ChatbotD -0.0169 0.159 432 0.05031106

4 ChatbotA - ChatbotE -0.2196 0.159 432 0.10393932

5 ChatbotA - ChatbotF 0.2822 0.159 432 0.14019082

6 ChatbotB - ChatbotC 0.7963 0.161 432 0.68939175

7 ChatbotB - ChatbotD 0.2472 0.159 432 0.11873005

8 ChatbotB - ChatbotE 0.0445 0.159 432 0.05215915

9 ChatbotB - ChatbotF 0.5464 0.160 432 0.39150263

10 ChatbotC - ChatbotD -0.5491 0.160 432 0.39469710

11 ChatbotC - ChatbotE -0.7518 0.161 432 0.63947893

12 ChatbotC - ChatbotF -0.2499 0.159 432 0.12027770

13 ChatbotD - ChatbotE -0.2027 0.159 432 0.09580295

14 ChatbotD - ChatbotF 0.2992 0.159 432 0.15170868

15 ChatbotE - ChatbotF 0.5018 0.160 432 0.33996364

## Supplemental Table S4 – Pairwise comparisons (inc. Cohen’s d effect sizes) for TestDay in the ANOVA model for Experiment 1.

Pairwise comparisons for TestDay

$emmeans

TestDay emmean SE df lower.CL upper.CL

Test1 1.03 0.0382 432 0.960 1.11

Test2 1.04 0.0382 432 0.964 1.11

Results are averaged over the levels of: ChatbotID, Domain, Prompt_type

Confidence level used: 0.95

$contrasts

contrast estimate SE df t.ratio p.value

Test1 - Test2 -0.00427 0.0541 432 -0.079 0.9371

Results are averaged over the levels of: ChatbotID, Domain, Prompt_type

Since 'object' is a list, we are using the contrasts already present.

Cohen's d effect sizes for TestDay comparisons

contrast effect.size SE df lower.CL upper.CL

(Test1 - Test2) -0.00724 0.0917 432 -0.188 0.173

Results are averaged over the levels of: ChatbotID, Domain, Prompt_type

sigma used for effect sizes: 0.5894

Confidence level used: 0.95

## Supplemental Table S5 – Pairwise comparisons (inc. Cohen’s d effect sizes) for Domain in the ANOVA model for Experiment 1.

Pairwise comparisons for Domain

$emmeans

Domain emmean SE df lower.CL upper.CL

Racing 1.05 0.0363 432 0.977 1.12

Training 1.03 0.0401 432 0.947 1.10

Results are averaged over the levels of: ChatbotID, TestDay, Prompt_type

Confidence level used: 0.95

$contrasts

contrast estimate SE df t.ratio p.value

Racing - Training 0.023 0.0541 432 0.426 0.6707

Results are averaged over the levels of: ChatbotID, TestDay, Prompt_type

Since 'object' is a list, we are using the contrasts already present.

Cohen's d effect sizes for Domain comparisons

contrast effect.size SE df lower.CL upper.CL

(Racing - Training) 0.039 0.0918 432 -0.141 0.219

Results are averaged over the levels of: ChatbotID, TestDay, Prompt_type

sigma used for effect sizes: 0.5894

Confidence level used: 0.95

## Supplemental Table S6 – Pairwise comparisons (inc. Cohen’s d effect sizes) for PromptType in the ANOVA model for Experiment 1.

Pairwise comparisons for Prompt_type

$emmeans

Prompt_type emmean SE df lower.CL upper.CL

DetailedPrompt 1.04 0.0382 432 0.968 1.12

SimplePrompt 1.03 0.0382 432 0.956 1.11

Results are averaged over the levels of: ChatbotID, TestDay, Domain

Confidence level used: 0.95

$contrasts

contrast estimate SE df t.ratio p.value

DetailedPrompt - SimplePrompt 0.0121 0.0541 432 0.224 0.8230

Results are averaged over the levels of: ChatbotID, TestDay, Domain

Since 'object' is a list, we are using the contrasts already present.

Cohen's d effect sizes for Prompt_type comparisons

contrast effect.size SE df lower.CL upper.CL

(DetailedPrompt - SimplePrompt) 0.0205 0.0917 432 -0.16 0.201

Results are averaged over the levels of: ChatbotID, TestDay, Domain

sigma used for effect sizes: 0.5894

Confidence level used: 0.95

## Supplemental Table S7 – Pairwise comparisons (inc. Cohen’s d effect sizes) for the ChatbotID * Prompt_type interaction in the ANOVA model for Experiment 1.

Pairwise comparisons for ChatbotID * Prompt_type interaction

$emmeans

ChatbotID Prompt_type emmean SE df lower.CL upper.CL

ChatbotA DetailedPrompt 1.067 0.0937 432 0.883 1.251

ChatbotB DetailedPrompt 1.168 0.0937 432 0.983 1.352

ChatbotC DetailedPrompt 0.872 0.0937 432 0.688 1.056

ChatbotD DetailedPrompt 0.996 0.0937 432 0.812 1.180

ChatbotE DetailedPrompt 1.128 0.0937 432 0.943 1.312

ChatbotF DetailedPrompt 1.028 0.0937 432 0.844 1.212

ChatbotA SimplePrompt 1.068 0.0937 432 0.884 1.252

ChatbotB SimplePrompt 1.279 0.0937 432 1.095 1.464

ChatbotC SimplePrompt 0.636 0.0937 432 0.452 0.820

ChatbotD SimplePrompt 1.160 0.0937 432 0.976 1.344

ChatbotE SimplePrompt 1.267 0.0937 432 1.083 1.451

ChatbotF SimplePrompt 0.775 0.0937 432 0.591 0.959

Results are averaged over the levels of: TestDay, Domain

Confidence level used: 0.95

$contrasts

contrast estimate SE df t.ratio

ChatbotA DetailedPrompt - ChatbotB DetailedPrompt -0.100145 0.132 432 -0.756

ChatbotA DetailedPrompt - ChatbotC DetailedPrompt 0.195128 0.132 432 1.473

ChatbotA DetailedPrompt - ChatbotD DetailedPrompt 0.071727 0.132 432 0.541

ChatbotA DetailedPrompt - ChatbotE DetailedPrompt -0.060198 0.132 432 -0.454

ChatbotA DetailedPrompt - ChatbotF DetailedPrompt 0.039480 0.132 432 0.298

ChatbotA DetailedPrompt - ChatbotA SimplePrompt -0.000846 0.132 432 -0.006

ChatbotA DetailedPrompt - ChatbotB SimplePrompt -0.212085 0.132 432 -1.601

ChatbotA DetailedPrompt - ChatbotC SimplePrompt 0.431321 0.132 432 3.256

ChatbotA DetailedPrompt - ChatbotD SimplePrompt -0.092550 0.132 432 -0.699

ChatbotA DetailedPrompt - ChatbotE SimplePrompt -0.199554 0.132 432 -1.507

ChatbotA DetailedPrompt - ChatbotF SimplePrompt 0.292342 0.132 432 2.207

ChatbotB DetailedPrompt - ChatbotC DetailedPrompt 0.295273 0.132 432 2.229

ChatbotB DetailedPrompt - ChatbotD DetailedPrompt 0.171872 0.132 432 1.298

ChatbotB DetailedPrompt - ChatbotE DetailedPrompt 0.039947 0.132 432 0.302

ChatbotB DetailedPrompt - ChatbotF DetailedPrompt 0.139625 0.132 432 1.054

ChatbotB DetailedPrompt - ChatbotA SimplePrompt 0.099299 0.132 432 0.750

ChatbotB DetailedPrompt - ChatbotB SimplePrompt -0.111940 0.132 432 -0.845

ChatbotB DetailedPrompt - ChatbotC SimplePrompt 0.531466 0.132 432 4.012

ChatbotB DetailedPrompt - ChatbotD SimplePrompt 0.007595 0.132 432 0.057

ChatbotB DetailedPrompt - ChatbotE SimplePrompt -0.099409 0.132 432 -0.750

ChatbotB DetailedPrompt - ChatbotF SimplePrompt 0.392487 0.132 432 2.963

ChatbotC DetailedPrompt - ChatbotD DetailedPrompt -0.123401 0.132 432 -0.932

ChatbotC DetailedPrompt - ChatbotE DetailedPrompt -0.255327 0.132 432 -1.928

ChatbotC DetailedPrompt - ChatbotF DetailedPrompt -0.155648 0.132 432 -1.175

ChatbotC DetailedPrompt - ChatbotA SimplePrompt -0.195974 0.132 432 -1.479

ChatbotC DetailedPrompt - ChatbotB SimplePrompt -0.407213 0.132 432 -3.074

ChatbotC DetailedPrompt - ChatbotC SimplePrompt 0.236193 0.132 432 1.783

ChatbotC DetailedPrompt - ChatbotD SimplePrompt -0.287679 0.132 432 -2.172

ChatbotC DetailedPrompt - ChatbotE SimplePrompt -0.394683 0.132 432 -2.980

ChatbotC DetailedPrompt - ChatbotF SimplePrompt 0.097214 0.132 432 0.734

ChatbotD DetailedPrompt - ChatbotE DetailedPrompt -0.131925 0.132 432 -0.996

ChatbotD DetailedPrompt - ChatbotF DetailedPrompt -0.032247 0.132 432 -0.243

ChatbotD DetailedPrompt - ChatbotA SimplePrompt -0.072573 0.132 432 -0.548

ChatbotD DetailedPrompt - ChatbotB SimplePrompt -0.283811 0.132 432 -2.143

ChatbotD DetailedPrompt - ChatbotC SimplePrompt 0.359594 0.132 432 2.715

ChatbotD DetailedPrompt - ChatbotD SimplePrompt -0.164277 0.132 432 -1.240

ChatbotD DetailedPrompt - ChatbotE SimplePrompt -0.271281 0.132 432 -2.048

ChatbotD DetailedPrompt - ChatbotF SimplePrompt 0.220615 0.132 432 1.666

ChatbotE DetailedPrompt - ChatbotF DetailedPrompt 0.099679 0.132 432 0.753

ChatbotE DetailedPrompt - ChatbotA SimplePrompt 0.059352 0.132 432 0.448

ChatbotE DetailedPrompt - ChatbotB SimplePrompt -0.151886 0.132 432 -1.147

ChatbotE DetailedPrompt - ChatbotC SimplePrompt 0.491520 0.132 432 3.711

ChatbotE DetailedPrompt - ChatbotD SimplePrompt -0.032352 0.132 432 -0.244

ChatbotE DetailedPrompt - ChatbotE SimplePrompt -0.139356 0.132 432 -1.052

ChatbotE DetailedPrompt - ChatbotF SimplePrompt 0.352540 0.132 432 2.661

ChatbotF DetailedPrompt - ChatbotA SimplePrompt -0.040326 0.132 432 -0.304

ChatbotF DetailedPrompt - ChatbotB SimplePrompt -0.251565 0.132 432 -1.899

ChatbotF DetailedPrompt - ChatbotC SimplePrompt 0.391841 0.132 432 2.958

ChatbotF DetailedPrompt - ChatbotD SimplePrompt -0.132030 0.132 432 -0.997

ChatbotF DetailedPrompt - ChatbotE SimplePrompt -0.239035 0.132 432 -1.805

ChatbotF DetailedPrompt - ChatbotF SimplePrompt 0.252862 0.132 432 1.909

ChatbotA SimplePrompt - ChatbotB SimplePrompt -0.211239 0.132 432 -1.595

ChatbotA SimplePrompt - ChatbotC SimplePrompt 0.432167 0.132 432 3.263

ChatbotA SimplePrompt - ChatbotD SimplePrompt -0.091704 0.132 432 -0.692

ChatbotA SimplePrompt - ChatbotE SimplePrompt -0.198708 0.132 432 -1.500

ChatbotA SimplePrompt - ChatbotF SimplePrompt 0.293188 0.132 432 2.213

ChatbotB SimplePrompt - ChatbotC SimplePrompt 0.643406 0.132 432 4.857

ChatbotB SimplePrompt - ChatbotD SimplePrompt 0.119534 0.132 432 0.902

ChatbotB SimplePrompt - ChatbotE SimplePrompt 0.012530 0.132 432 0.095

ChatbotB SimplePrompt - ChatbotF SimplePrompt 0.504427 0.132 432 3.808

ChatbotC SimplePrompt - ChatbotD SimplePrompt -0.523872 0.132 432 -3.955

ChatbotC SimplePrompt - ChatbotE SimplePrompt -0.630876 0.132 432 -4.763

ChatbotC SimplePrompt - ChatbotF SimplePrompt -0.138979 0.132 432 -1.049

ChatbotD SimplePrompt - ChatbotE SimplePrompt -0.107004 0.132 432 -0.808

ChatbotD SimplePrompt - ChatbotF SimplePrompt 0.384892 0.132 432 2.906

ChatbotE SimplePrompt - ChatbotF SimplePrompt 0.491896 0.132 432 3.714

p.value

0.9998

0.9471

1.0000

1.0000

1.0000

1.0000

0.9081

0.0547

0.9999

0.9383

0.5454

0.5293

0.9792

1.0000

0.9963

0.9998

0.9995

0.0040

1.0000

0.9998

0.1233

0.9988

0.7414

0.9906

0.9455

0.0919

0.8265

0.5711

0.1182

0.9999

0.9977

1.0000

1.0000

0.5923

0.2225

0.9854

0.6600

0.8828

0.9998

1.0000

0.9923

0.0124

1.0000

0.9963

0.2494

1.0000

0.7593

0.1249

0.9977

0.8149

0.7532

0.9104

0.0537

0.9999

0.9401

0.5408

0.0001

0.9991

1.0000

0.0087

0.0050

0.0002

0.9964

0.9997

0.1425

0.0123

Results are averaged over the levels of: TestDay, Domain

P value adjustment: tukey method for comparing a family of 12 estimates

Since 'object' is a list, we are using the contrasts already present.

Cohen's d effect sizes for ChatbotID * Prompt_type comparisons

contrast effect.size SE df

(ChatbotA DetailedPrompt - ChatbotB DetailedPrompt) -0.16991 0.225 432

(ChatbotA DetailedPrompt - ChatbotC DetailedPrompt) 0.33106 0.225 432

(ChatbotA DetailedPrompt - ChatbotD DetailedPrompt) 0.12169 0.225 432

(ChatbotA DetailedPrompt - ChatbotE DetailedPrompt) -0.10213 0.225 432

(ChatbotA DetailedPrompt - ChatbotF DetailedPrompt) 0.06698 0.225 432

(ChatbotA DetailedPrompt - ChatbotA SimplePrompt) -0.00144 0.225 432

(ChatbotA DetailedPrompt - ChatbotB SimplePrompt) -0.35982 0.225 432

(ChatbotA DetailedPrompt - ChatbotC SimplePrompt) 0.73178 0.226 432

(ChatbotA DetailedPrompt - ChatbotD SimplePrompt) -0.15702 0.225 432

(ChatbotA DetailedPrompt - ChatbotE SimplePrompt) -0.33856 0.225 432

(ChatbotA DetailedPrompt - ChatbotF SimplePrompt) 0.49599 0.225 432

(ChatbotB DetailedPrompt - ChatbotC DetailedPrompt) 0.50096 0.225 432

(ChatbotB DetailedPrompt - ChatbotD DetailedPrompt) 0.29160 0.225 432

(ChatbotB DetailedPrompt - ChatbotE DetailedPrompt) 0.06777 0.225 432

(ChatbotB DetailedPrompt - ChatbotF DetailedPrompt) 0.23689 0.225 432

(ChatbotB DetailedPrompt - ChatbotA SimplePrompt) 0.16847 0.225 432

(ChatbotB DetailedPrompt - ChatbotB SimplePrompt) -0.18992 0.225 432

(ChatbotB DetailedPrompt - ChatbotC SimplePrompt) 0.90169 0.227 432

(ChatbotB DetailedPrompt - ChatbotD SimplePrompt) 0.01289 0.225 432

(ChatbotB DetailedPrompt - ChatbotE SimplePrompt) -0.16866 0.225 432

(ChatbotB DetailedPrompt - ChatbotF SimplePrompt) 0.66590 0.226 432

(ChatbotC DetailedPrompt - ChatbotD DetailedPrompt) -0.20936 0.225 432

(ChatbotC DetailedPrompt - ChatbotE DetailedPrompt) -0.43319 0.225 432

(ChatbotC DetailedPrompt - ChatbotF DetailedPrompt) -0.26407 0.225 432

(ChatbotC DetailedPrompt - ChatbotA SimplePrompt) -0.33249 0.225 432

(ChatbotC DetailedPrompt - ChatbotB SimplePrompt) -0.69088 0.226 432

(ChatbotC DetailedPrompt - ChatbotC SimplePrompt) 0.40073 0.225 432

(ChatbotC DetailedPrompt - ChatbotD SimplePrompt) -0.48808 0.225 432

(ChatbotC DetailedPrompt - ChatbotE SimplePrompt) -0.66962 0.226 432

(ChatbotC DetailedPrompt - ChatbotF SimplePrompt) 0.16493 0.225 432

(ChatbotD DetailedPrompt - ChatbotE DetailedPrompt) -0.22382 0.225 432

(ChatbotD DetailedPrompt - ChatbotF DetailedPrompt) -0.05471 0.225 432

(ChatbotD DetailedPrompt - ChatbotA SimplePrompt) -0.12313 0.225 432

(ChatbotD DetailedPrompt - ChatbotB SimplePrompt) -0.48152 0.225 432

(ChatbotD DetailedPrompt - ChatbotC SimplePrompt) 0.61009 0.226 432

(ChatbotD DetailedPrompt - ChatbotD SimplePrompt) -0.27871 0.225 432

(ChatbotD DetailedPrompt - ChatbotE SimplePrompt) -0.46026 0.225 432

(ChatbotD DetailedPrompt - ChatbotF SimplePrompt) 0.37430 0.225 432

(ChatbotE DetailedPrompt - ChatbotF DetailedPrompt) 0.16912 0.225 432

(ChatbotE DetailedPrompt - ChatbotA SimplePrompt) 0.10070 0.225 432

(ChatbotE DetailedPrompt - ChatbotB SimplePrompt) -0.25769 0.225 432

(ChatbotE DetailedPrompt - ChatbotC SimplePrompt) 0.83391 0.227 432

(ChatbotE DetailedPrompt - ChatbotD SimplePrompt) -0.05489 0.225 432

(ChatbotE DetailedPrompt - ChatbotE SimplePrompt) -0.23643 0.225 432

(ChatbotE DetailedPrompt - ChatbotF SimplePrompt) 0.59812 0.226 432

(ChatbotF DetailedPrompt - ChatbotA SimplePrompt) -0.06842 0.225 432

(ChatbotF DetailedPrompt - ChatbotB SimplePrompt) -0.42681 0.225 432

(ChatbotF DetailedPrompt - ChatbotC SimplePrompt) 0.66480 0.226 432

(ChatbotF DetailedPrompt - ChatbotD SimplePrompt) -0.22400 0.225 432

(ChatbotF DetailedPrompt - ChatbotE SimplePrompt) -0.40555 0.225 432

(ChatbotF DetailedPrompt - ChatbotF SimplePrompt) 0.42901 0.225 432

(ChatbotA SimplePrompt - ChatbotB SimplePrompt) -0.35839 0.225 432

(ChatbotA SimplePrompt - ChatbotC SimplePrompt) 0.73322 0.226 432

(ChatbotA SimplePrompt - ChatbotD SimplePrompt) -0.15559 0.225 432

(ChatbotA SimplePrompt - ChatbotE SimplePrompt) -0.33713 0.225 432

(ChatbotA SimplePrompt - ChatbotF SimplePrompt) 0.49742 0.225 432

(ChatbotB SimplePrompt - ChatbotC SimplePrompt) 1.09160 0.228 432

(ChatbotB SimplePrompt - ChatbotD SimplePrompt) 0.20280 0.225 432

(ChatbotB SimplePrompt - ChatbotE SimplePrompt) 0.02126 0.225 432

(ChatbotB SimplePrompt - ChatbotF SimplePrompt) 0.85581 0.227 432

(ChatbotC SimplePrompt - ChatbotD SimplePrompt) -0.88880 0.227 432

(ChatbotC SimplePrompt - ChatbotE SimplePrompt) -1.07035 0.228 432

(ChatbotC SimplePrompt - ChatbotF SimplePrompt) -0.23579 0.225 432

(ChatbotD SimplePrompt - ChatbotE SimplePrompt) -0.18154 0.225 432

(ChatbotD SimplePrompt - ChatbotF SimplePrompt) 0.65301 0.226 432

(ChatbotE SimplePrompt - ChatbotF SimplePrompt) 0.83455 0.227 432

lower.CL upper.CL

-0.6118 0.27195

-0.1112 0.77332

-0.3201 0.56347

-0.5439 0.33963

-0.3747 0.50871

-0.4431 0.44027

-0.8022 0.08254

0.2874 1.17619

-0.5989 0.28481

-0.7809 0.10372

0.0530 0.93894

0.0580 0.94394

-0.1505 0.73374

-0.3740 0.50950

-0.2051 0.67888

-0.2734 0.61032

-0.6318 0.25197

0.4559 1.34749

-0.4288 0.45459

-0.6105 0.27319

0.2220 1.10984

-0.6513 0.23256

-0.8758 0.00947

-0.7061 0.17799

-0.7748 0.10977

-1.1350 -0.24676

-0.0418 0.84324

-0.9310 -0.04517

-1.1136 -0.22565

-0.2769 0.60678

-0.6658 0.21814

-0.4964 0.38701

-0.5649 0.31866

-0.9244 -0.03864

0.1665 1.05368

-0.7208 0.16339

-0.9030 -0.01748

-0.0681 0.81671

-0.2727 0.61097

-0.3411 0.54246

-0.6997 0.18435

0.3887 1.27913

-0.4966 0.38683

-0.6784 0.20556

0.1546 1.04164

-0.5101 0.37331

-0.8694 0.01582

0.2209 1.10874

-0.6660 0.21796

-0.8481 0.03699

-0.0136 0.87164

-0.8007 0.08397

0.2888 1.17764

-0.5974 0.28624

-0.7794 0.10515

0.0545 0.94038

0.6439 1.53930

-0.2391 0.64472

-0.4204 0.46297

0.4104 1.30121

-1.3345 -0.44312

-1.5178 -0.62288

-0.6778 0.20620

-0.6234 0.26033

0.2092 1.09687

0.3893 1.27977

Results are averaged over the levels of: TestDay, Domain

sigma used for effect sizes: 0.5894

Confidence level used: 0.95

## Supplemental Table S8 – The LMM model summary table for Experiment 2.

LMM_Model_1

Generalized linear mixed model fit by maximum likelihood (Laplace

Approximation) [glmerMod]

Family: binomial ( logit )

Formula: Correct_answer ~ ChatbotID + TestDay + ExamDomain + (1 | ChatbotID)

Data: Experiment2_forLMM

AIC BIC logLik deviance df.resid

1338.7 1390.6 -659.4 1318.7 1322

Scaled residuals:

Min 1Q Median 3Q Max

-3.6305 0.2754 0.4306 0.5340 0.9945

Random effects:

Groups Name Variance Std.Dev.

ChatbotID (Intercept) 0 0

Number of obs: 1332, groups: ChatbotID, 6

Fixed effects:

Estimate Std. Error z value Pr(>|z|)

(Intercept) 1.33857 0.18012 7.432 1.07e-13 ***

ChatbotIDChatbotB 0.43024 0.22633 1.901 0.057308 .

ChatbotIDChatbotC -0.15768 0.21240 -0.742 0.457864

ChatbotIDChatbotD 0.04673 0.21619 0.216 0.828864

ChatbotIDChatbotE 0.94692 0.24756 3.825 0.000131 ***

ChatbotIDChatbotF 1.24019 0.26444 4.690 2.73e-06 ***

TestDayTest2 -0.08384 0.13654 -0.614 0.539158

ExamDomainB -1.08609 0.14039 -7.736 1.02e-14 ***

ExamDomainC 0.16862 0.33053 0.510 0.609948

---

Signif. codes: 0 ‘***’ 0.001 ‘**’ 0.01 ‘*’ 0.05 ‘.’ 0.1 ‘ ’ 1

Correlation of Fixed Effects:

(Intr) ChIDCB ChIDCC ChIDCD ChIDCE ChIDCF TstDT2 ExmDmB

ChtbtIDChtB -0.558

ChtbtIDChtC -0.610 0.481

ChtbtIDChtD -0.594 0.473 0.504

ChtbtIDChtE -0.502 0.415 0.440 0.433

ChtbtIDChtF -0.466 0.389 0.412 0.405 0.357

TestDayTst2 -0.388 -0.002 0.001 0.000 -0.004 -0.004

ExamDomainB -0.361 -0.027 0.012 -0.003 -0.049 -0.055 0.008

ExamDomainC -0.160 0.001 -0.001 0.000 0.002 0.002 0.000 0.204

## Supplemental Table S9 – Pairwise comparisons for ChatbotID in the LMM model for Experiment 2.

Pairwise comparisons for ChatbotID for LMM_Model_1

$emmeans

A summary_emm: 6 × 6

ChatbotID emmean SE df asymp.LCL asymp.UCL

<fct> <dbl> <dbl> <dbl> <dbl> <dbl>

1 ChatbotA 0.9908193 0.1778363 Inf 0.6422667 1.339372

2 ChatbotB 1.4210625 0.1917260 Inf 1.0452864 1.796839

3 ChatbotC 0.8331429 0.1741174 Inf 0.4918791 1.174407

4 ChatbotD 1.0375501 0.1790732 Inf 0.6865730 1.388527

5 ChatbotE 1.9377383 0.2168610 Inf 1.5126986 2.362778

6 ChatbotF 2.2310101 0.2361240 Inf 1.7682155 2.693805

$contrasts

A summary_emm: 15 × 6

contrast estimate SE df z.ratio p.value

<chr> <dbl> <dbl> <dbl> <dbl> <dbl>

1 ChatbotA - ChatbotB -0.43024313 0.2263299 Inf -1.9009560 4.014438e-01

2 ChatbotA - ChatbotC 0.15767645 0.2123962 Inf 0.7423695 9.766184e-01

3 ChatbotA - ChatbotD -0.04673076 0.2161874 Inf -0.2161586 9.999356e-01

4 ChatbotA - ChatbotE -0.94691897 0.2475639 Inf -3.8249483 1.819482e-03

5 ChatbotA - ChatbotF -1.24019074 0.2644395 Inf -4.6898840 4.022054e-05

6 ChatbotB - ChatbotC 0.58791958 0.2237114 Inf 2.6280269 9.055324e-02

7 ChatbotB - ChatbotD 0.38351237 0.2272211 Inf 1.6878375 5.398434e-01

8 ChatbotB - ChatbotE -0.51667584 0.2569678 Inf -2.0106634 3.360666e-01

9 ChatbotB - ChatbotF -0.80994761 0.2732000 Inf -2.9646694 3.586393e-02

10 ChatbotC - ChatbotD -0.20440721 0.2133770 Inf -0.9579629 9.310438e-01

11 ChatbotC - ChatbotE -1.10459542 0.2452369 Inf -4.5041974 9.733705e-05

12 ChatbotC - ChatbotF -1.39786719 0.2622859 Inf -5.3295560 1.467879e-06

13 ChatbotD - ChatbotE -0.90018821 0.2483623 Inf -3.6244966 3.928722e-03

14 ChatbotD - ChatbotF -1.19345998 0.2651776 Inf -4.5006059 9.898092e-05

15 ChatbotE - ChatbotF -0.29327177 0.2907223 Inf -1.0087696 9.152195e-01

## Supplemental Table S10 – Pairwise comparisons for ExamDomain in the LMM model for Experiment 2.

Pairwise comparisons for ExamDomain for LMM_Model_1

$emmeans

A summary_emm: 3 × 6

ExamDomain emmean SE df asymp.LCL asymp.UCL

<fct> <dbl> <dbl> <dbl> <dbl> <dbl>

1 A 1.7143774 0.1004651 Inf 1.5174695 1.911285

2 B 0.6282873 0.1013206 Inf 0.4297025 0.826872

3 C 1.8829969 0.3169311 Inf 1.2618233 2.504171

$contrasts

A summary_emm: 3 × 6

contrast estimate SE df z.ratio p.value

<chr> <dbl> <dbl> <dbl> <dbl> <dbl>

1 A - B 1.0860901 0.1403864 Inf 7.736433 4.873879e-14

2 A - C -0.1686196 0.3305307 Inf -0.510148 8.664029e-01

3 B - C -1.2547097 0.3317118 Inf -3.782529 4.558251e-04

## Supplemental Table S11 – Pairwise comparisons for TestDay in the LMM model for Experiment 2.

Pairwise comparisons for TestDay for LMM_Model_1

$emmeans

A summary_emm: 2 × 6

TestDay emmean SE df asymp.LCL asymp.UCL

<fct> <dbl> <dbl> <dbl> <dbl> <dbl>

1 Test1 1.450476 0.1363650 Inf 1.183206 1.717747

2 Test2 1.366631 0.1347319 Inf 1.102562 1.630701

$contrasts

A summary_emm: 1 × 6

contrast estimate SE df z.ratio p.value

<chr> <dbl> <dbl> <dbl> <dbl> <dbl>

1 Test1 - Test2 0.08384489 0.136536 Inf 0.6140864 0.5391583

## Supplemental Table S12 – Effect sizes (r) and power for ChatbotID in the LMM model for Experiment 2.

Contrasts table for ChatbotID for LMM_Model_1

contrast estimate SE df z.ratio p.value r

ChatbotA - ChatbotB -0.4302431 0.2263299 Inf -1.901 0.4014 -0.8850147

ChatbotA - ChatbotC 0.1576765 0.2123962 Inf 0.742 0.9766 0.5960716

ChatbotA - ChatbotD -0.0467308 0.2161874 Inf -0.216 0.9999 -0.2112790

ChatbotA - ChatbotE -0.9469190 0.2475639 Inf -3.825 0.0018 -0.9674820

ChatbotA - ChatbotF -1.2401907 0.2644395 Inf -4.690 <.0001 -0.9780145

ChatbotB - ChatbotC 0.5879196 0.2237114 Inf 2.628 0.0906 0.9346241

ChatbotB - ChatbotD 0.3835124 0.2272212 Inf 1.688 0.5398 0.8603362

ChatbotB - ChatbotE -0.5166758 0.2569678 Inf -2.011 0.3361 -0.8953749

ChatbotB - ChatbotF -0.8099476 0.2732000 Inf -2.965 0.0359 -0.9475480

ChatbotC - ChatbotD -0.2044072 0.2133770 Inf -0.958 0.9310 -0.6917659

ChatbotC - ChatbotE -1.1045954 0.2452369 Inf -4.504 0.0001 -0.9762299

ChatbotC - ChatbotF -1.3978672 0.2622859 Inf -5.330 <.0001 -0.9828485

ChatbotD - ChatbotE -0.9001882 0.2483623 Inf -3.624 0.0039 -0.9639831

ChatbotD - ChatbotF -1.1934600 0.2651776 Inf -4.501 0.0001 -0.9761932

ChatbotE - ChatbotF -0.2932718 0.2907223 Inf -1.009 0.9152 -0.7101870

SE_r r_CI_lower r_CI_upper Power

0.5773503 -0.9873902 -0.2606792 0.4765293

0.5773503 -0.4174294 0.9487025 0.1151318

0.5773503 -0.8731335 0.7245211 0.0553696

0.5773503 -0.9965676 -0.7257798 0.9689082

0.5773503 -0.9976904 -0.8069160 0.9968325

0.5773503 0.5095714 0.9929947 0.7479555

0.5773503 0.1616016 0.9845032 0.3928946

0.5773503 -0.9885822 -0.3065994 0.5202533

0.5773503 -0.9944129 -0.5886566 0.8424811

0.5773503 -0.9628023 0.2731570 0.1599334

0.5773503 -0.9975009 -0.7926984 0.9945241

0.5773503 -0.9982022 -0.8464498 0.9996236

0.5773503 -0.9961922 -0.7002368 0.9519970

0.5773503 -0.9974970 -0.7924089 0.9944675

0.5773503 -0.9653571 0.2393132 0.1722480

Results are averaged over the levels of: TestDay, ExamDomain

Results are given on the log odds ratio (not the response) scale.

P value adjustment: tukey method for comparing a family of 6 estimates

## Supplemental Table S13 – Effect sizes (r) and power for ExamDomain in the LMM model for Experiment 2.

Contrasts table for ExamDomain for LMM_Model_1

contrast estimate SE df z.ratio p.value r SE_r

A - B 1.0860901 0.1403864 Inf 7.736 <.0001 0.9917494 0.2581989

A - C -0.1686196 0.3305307 Inf -0.510 0.8664 -0.4544307 0.2581989

B - C -1.2547097 0.3317118 Inf -3.783 0.0005 -0.9667847 0.2581989

r_CI_lower r_CI_upper Power

0.9774614 0.9969935 1.0000000

-0.7600527 0.0157985 0.0803085

-0.9877992 -0.9111920 0.9658154

Results are averaged over the levels of: ChatbotID, TestDay

Results are given on the log odds ratio (not the response) scale.

P value adjustment: tukey method for comparing a family of 3 estimates

## Supplemental Table S14 – Effect sizes (r) and power for TestDay in the LMM model for Experiment 2.

Contrasts table for TestDay for LMM_Model_1

contrast estimate SE df z.ratio p.value r SE_r

Test1 - Test2 0.08384489 0.136536 Inf 0.614 0.5392 0.5232948 0.3333333

r_CI_lower r_CI_upper Power

-0.07234049 0.8437932 0.09419677

Results are averaged over the levels of: ChatbotID, ExamDomain

Results are given on the log odds ratio (not the response) scale.
