# Supplementary material for: The sports nutrition knowledge of large language model (LLM) artificial intelligence (AI) chatbots: An assessment of accuracy, completeness, clarity, quality of evidence, and test-retest reliability
Source: PLoS One. 2025 Jun 13;20(6):e0325982. doi: 10.1371/journal.pone.0325982 (PMC12165421; doi:10.1371/journal.pone.0325982)
Supplement: S2 File — (DOCX) [file pone.0325982.s002.docx]

## **Supplemental file: METRICS checklist**

| Item | Issues considered | Page(s), Table(s), and Figure(s) that address the issue |
| --- | --- | --- |
| Model | What is the model of the generative AI tool used for generating content, and what are the exact settings for each tool? | 8, 13, Table 2 |
| Evaluation | What is the exact approach used to evaluate the content generated by the generative AI-based model and is it an objective or subjective evaluation? | 9-12, 14 |
| Timing | When is the generative AI model tested exactly and what are the duration and timing of testing? | 5 |
| Transparency | How transparent are the data sources used to generate queries for the generative AI-based model? | 17 |
| Range | What is the range of topics tested and are they intersubject or intrasubject with variability in different subjects? | 6-8, 13, Table 1 |
| Randomisation | Was the process of selecting the topics to be tested on the generative AI-based model randomized? | 13 |
| Individual | Is there any individual subjective involvement in generative AI content evaluation? If so, did the authors describe the details in full? | 9-12 |
| Count | What is the count of queries executed (sample size)? | 8, 13, Table 2, Table 3 |
| Specificity | How specific are the exact prompts used? Were those exact prompts provided fully? Did the authors consider the feedback and learning loops? How specific are the language and cultural issues considered in the generative AI model? | 6-7, 13, Table 1 |
