# Supplementary material for: The sports nutrition knowledge of large language model (LLM) artificial intelligence (AI) chatbots: An assessment of accuracy, completeness, clarity, quality of evidence, and test-retest reliability
Source: PLoS One. 2025 Jun 13;20(6):e0325982. doi: 10.1371/journal.pone.0325982 (PMC12165421; doi:10.1371/journal.pone.0325982)
Supplement: S3 File — (DOCX) [file pone.0325982.s003.docx]

## **Supplemental file: Guidelines for Reporting Reliability and Agreement Studies (GRRAS) checklist**

| Section | Item | Page(s) on which the item is addressed |
| --- | --- | --- |
| Title/Abstract | Identify in the title or abstract that interrater/intrarater reliability or agreement was investigated. | 2 |
| Introduction | Name and describe the diagnostic or measurement device of interest explicitly. | 4-5, 9-12, 14 |
|  | Specify the subject population of interest. | 4-5, Table 2 |
|  | Specify the rater population of interest (if applicable). | 4-5, 10, 14 |
|  | Describe what is already known about reliability and agreement and provide a rationale for the study (if applicable). | 4-5 |
| Methods | Explain how the sample size was chosen. State the determined number of raters, subjects/objects, and replicate observations. | 14 |
|  | Describe the sampling method. | 14-16 |
|  | Describe the measurement/rating process (e.g. time interval between repeated measurements, availability of clinical information, blinding). | 8, 13 |
|  | State whether measurements/ratings were conducted independently. | 8, 13 |
|  | Describe the statistical analysis. | 14-16 |
| Results | State the actual number of raters and subjects/objects which were included and the number of replicate observations which were conducted. | 8, 13 |
|  | Describe the sample characteristics of raters and subjects (e.g. training, experience). | 9-12, 14 |
|  | Report estimates of reliability and agreement including measures of statistical uncertainty. | 17, 22 |
| Discussion | Discuss the practical relevance of results. | 25-30 |
| Auxiliary material | Provide detailed results if possible (e.g. online). | 17, 18 |
