# Supplementary material for: The sports nutrition knowledge of large language model (LLM) artificial intelligence (AI) chatbots: An assessment of accuracy, completeness, clarity, quality of evidence, and test-retest reliability
Source: PLoS One. 2025 Jun 13;20(6):e0325982. doi: 10.1371/journal.pone.0325982 (PMC12165421; doi:10.1371/journal.pone.0325982)
Supplement: Attachment — Submitted filename: Responses to Reviewers.docx [file pone.0325982.s005.docx]

# Response to Reviewers

## Editor’s comments:

**Thank you for your comments. We have addressed them point-by-point in bold blue font below.**

1. Please ensure that your manuscript meets PLOS ONE's style requirements, including those for file naming. The PLOS ONE style templates can be found at <https://journals.plos.org/plosone/s/file?id=wjVg/PLOSOne_formatting_sample_main_body.pdf> and <https://journals.plos.org/plosone/s/file?id=ba62/PLOSOne_formatting_sample_title_authors_affiliations.pdf>

**Our revised manuscript is formatted according to style requirements outlined in the weblinks.**

2. Thank you for stating the following in the Competing Interests section: [TS has given invited talks at societal conferences and university/pharmaceutical symposia for which the organisers paid for travel and accommodation; he has also received research money from publicly funded national research councils and medical charities, and private companies, including Novo Nordisk Foundation, AstraZeneca, Amylin, AP Møller Foundation, and Augustinus Foundation; and, he has consulted for Boost Treadmills, GU Energy, and Examine.com, and owns a consulting business, Blazon Scientific, and an endurance athlete education business, Veohtu. These companies have had no control over the research design, data analysis, or publication outcomes of this work. ML has given invited talks at societal conferences and university symposia and meetings for which the organisers paid for travel and accommodation; he has received research money from Augustinus Foundation, American College of Sports Medicine, and national research institutions; and, he has consulted for Zepp Health, Levels Health, GU Energy, and EAB labs, and has coached for Sharman Ultra Coaching. These companies have had no control over the research design, data analysis, or publication outcomes of this work. My Sports Dietitian provided a set of multiple-choice questions designed to resemble the Certified Specialist in Sports Dietetics (CSSD) board exam. Neither TPJS nor MJL have any financial relationships with My Sports Dietitian.]. We note that you received funding from a commercial source: [Novo Nordisk Foundation, AstraZeneca, Amylin, AP Møller Foundation, and Augustinus Foundation]. Please provide an amended Competing Interests Statement that explicitly states this commercial funder, along with any other relevant declarations relating to employment, consultancy, patents, products in development, marketed products, etc. Within this Competing Interests Statement, please confirm that this does not alter your adherence to all PLOS ONE policies on sharing data and materials by including the following statement: "This does not alter our adherence to PLOS ONE policies on sharing data and materials.” (as detailed online in our guide for authors <http://journals.plos.org/plosone/s/competing-interests>). If there are restrictions on sharing of data and/or materials, please state these. Please note that we cannot proceed with consideration of your article until this information has been declared. Please include your amended Competing Interests Statement within your cover letter. We will change the online submission form on your behalf.

**We have added this information to the manuscript and included the revised Competing Interests statement in our cover letter.**

3. Thank you for stating the following financial disclosure: [The work was funded by internal institutional funds from the Idaho College of Osteopathic Medicine. No external funds were received for this work.]. Please state what role the funders took in the study. If the funders had no role, please state: ""The funders had no role in study design, data collection and analysis, decision to publish, or preparation of the manuscript."" If this statement is not correct you must amend it as needed. Please include this amended Role of Funder statement in your cover letter; we will change the online submission form on your behalf.

**We have added this information to the manuscript and included this statement in our cover letter.**

## Reviewers' comments:

### **Reviewer 1**

**Thank you for your comments. We have addressed them point-by-point in bold blue font below.**

1. The “basic” and “advanced” versions of AI chatbots are clearly categorized. However, additional information should be provided about the version dates of the LLM models and the datasets they were trained on.

**We have added release date information to Table 2.**

**The model manufacturers do not provide precise details on which datasets their models are trained on, but they are all multimodal models, meaning they were trained on several datasets. Importantly, we did not fine-tune the models in any way; we added information to clarify that on lines 118 and 220.**

2. The simple and detailed prompts entered into the chatbots are clearly listed. However, is there a control mechanism on the update status of the chatbots or the impact of users’ past chat history? Please provide some information about this.

**Yes, there was a control mechanism for the update status of the chatbots: We input the test and retest prompts on October 7, 2024 and October 11, 2024, less than 1 week apart, to prevent model updates influencing changes in the output. No model updates were made in that timeframe; we added information to clarify that point on lines 121 and 233.**

**And, yes, we also considered the impact of users’ past chat history. For example, as explained in the *Prompt inputting* sections (lines 115 and 226), new chatbot accounts were created, the chatbots’ histories were deleted before each new prompt was entered, the chatbots’ memories were turned off, and a new chat was created each time a new prompt was entered.**

3. More information can be added about the data collection process and bias control.

**The original manuscript described the data collection process with a high level of detail. For example, the *Prompt inputting* sections (lines 125-129 and lines 244-247) describe how our independent prompter entered the prompts and collected and blinded the data. However, although the processes were described, their purpose (i.e., to minimise bias) was not explicitly stated. So, please see that we have added some text to those sections to clarify that these approaches were used to minimise bias.**

**Other approaches to minimise bias included:** **the independent ratings by the two investigators (Experiment 1: line 145-146), the use of an automated script to rate the exam questions (Experiment 2: line 251-252), the deletion of chatbot history (lines 122-124), not using chatbot customisations or plug-ins (lines 122-124), turning the chatbot’s memory off (lines 122-124), and creating a new chat each time a new prompt was entered (lines 122-124). We have added some text to lines 698-700 to clarify these points.**

**Furthermore, we used the intraclass correlation coefficient (ICC) to examine absolute agreement between the raters’ scores (lines 273-376), finding that the interrater reliability between the two raters was *good* (ICC = 0.893, 95% CI 0.869 to 0.912; F = 9.82, p < 0.001) (line 349). Additionally, we used the Kuder-Richardson 20 (KR-20) statistic to measure the internal consistency reliability of the exam (line 308-311), finding a KR-20 value of 0.895, indicating good internal reliability of the exam and suggesting that the exam questions were measuring the same underlying construct consistently and, thereby, effectively assessing the knowledge it was designed to measure (line 513-515).**

4. The time between two tests is very short (4 days: October 7, 2024 and October 11, 2024). Since AI models can be updated frequently, a longer time interval may be required for test-retest.

**If our research question was to examine the time course of how chatbot outputs change with time (i.e. with model development), then a longer time interval would have been prudent. However, the purpose of the test-retest approach in the context of our research question was to examine the test-retest reliability of information provided by chatbots. If a longer time interval was chosen, a chatbot model may have been updated (because AI models are updated frequently); consequently, any comparisons between dates would introduce bias and be unable to provide information regarding the reliability of a specific chatbot model.**

5. The study stated that the referees who evaluated the chatbot responses evaluated the outputs blindly. However: The evaluation criteria used by the referees were not clearly given. The criteria used by the referees for scoring should be explained in more detail and compared with other referees.

**The evaluation criteria are described and explained in detail on lines 133-215, 269-287, and 312-318 and complemented by the information provided in Table 3. These evaluation approaches have been used previously in similar diet/exercise investigations. For example, Haman *et al*, Papastratis *et al*, Sun *et al*, and Niszczota *et al* measured the accuracy of chatbot outputs, but they only used qualitative evaluations, not statistical analyses, to examine between-chatbot comparisons. Meanwhile,** **other studies have used Likert scales to compare different metrics between chatbots, including accuracy (Düking *et al*, Pugliese *et al*, Pérez-Guerrero *et al*, Goodman *et al*), completeness/comprehensiveness (Pugliese *et al*, Pérez-Guerrero *et al*, Goodman *et al*), clarity/coherence (Pérez-Guerrero *et al*, Goodman *et al*), evidence quality (Pérez-Guerrero *et al*), and test-retest reliability (Goodman *et al*), along with statistical tests to examine between-chatbot comparisons.**

**While we cited all these papers in the original manuscript, we did not explicitly indicate how these evaluation approaches had previously been used. We have added text to remedy that issue (lines 148-149).**

6. It should be detailed which types of nutrition questions each model is more successful in. For example, if Gemini uses more scientific references and Claude explains in more general terms, this should be emphasized.

**This information was provided on line 403 onwards, but we have added a little more detail on lines 404-406 and 412-416 to address your point and improve the clarity of the results.**

### **Reviewer 2**

The study was well-designed and the statistics are good. However, some points should be addressed.

**Thank you for your comments. We have responded point-by-point in bold blue text below.**

The first page of introduction may be deleted. Kindly focus on three elements of introduction: a. What is known about the topic? (Background). b. What is not known? (The research problem). c. Why the study was done? (Justification)

**These are general comments on how to write an introduction section. Our original introduction already contains clear information about the *background*, the *problem*, and the *justification*. We will not delete the first page.**

Model - What are the exact settings for each tool?

**Our original methods section already contains this information. Please see Table 2 and the *Prompt inputting* sections on lines 115 and 226.**

Evaluation - What is the exact approach used to evaluate the content generated by the generative AI-based model and is it an objective or subjective evaluation?

**Our original methods section already contains this information. Please see the *Rating chatbot performance* sections on lines 133-211 and 249-254. We have added info on lines 146 and 251 to specifically indicate whether the evaluations were objective or subjective.**

Explain in details the Randomisation - Was the process of selecting the topics to be tested on the generative AI-based model randomized?

**The process of selecting the topics to be tested on the generative AI-based model was not randomised because that was not how our study was designed. That said, our original methods section did provide information about randomisation of the order of the MCQ questions (line 233-234).**

Specificity is not clear, please clarify the following points:

How specific are the exact prompts used?

**The specific prompts are already clearly described in Table 1. However, we have added a sentence to clarify the prompts’ specificity to sports nutrition (lines 93 and 221).**

Were those exact prompts provided fully?

**Yes. The specific prompts are clearly described in Table 1.**

Did the authors consider the feedback and learning loops?

**Yes, as described in the *Prompt inputting* sections (lines 115 and 226), new chatbot accounts were created, the chatbots’ histories were deleted before each new prompt was entered, the chatbots’ memories were turned off, and a new chat was created each time a new prompt was entered.** **We used these practices to prevent feedback and learning loop effects; we have added a sentence to clarify that on lines 121 and 240.**

How specific are the language and cultural issues considered in the generative AI model?

**All prompts were written in English (added text on lines 94 and 221). We added a sentence about cultural issues on line 112.**

Discussion: The discussion section needs to be described scientifically. Kindly frame it along the following lines: i. Main findings of the present study. ii. Comparison with other studies. iii. Implication and explanation of findings. iv. Strengths and limitations. v. Conclusion, recommendation.

**These are general comments on how to write a discussion section. Our original discussion already contains all these aspects.**

### **Reviewer 3**

**Thank you for your comments. We have addressed them point-by-point in bolded blue font below.**

1. Title, Abstract and Introduction

• Lines 8 – 14 lacks specific details about the statistical methods used to assess chatbot performance. Also, statistical methods for assessing interrater reliability and chatbot accuracy should be mentioned.

**Thank you for spotting this omission. We have added text to address this point on lines 9 and 13.**

• Lines 24 – 25 could be extended to include suggestions or recommendations for improving chatbot reliability.

**Because the study was not designed to improve chatbot reliability in future versions, adding suggestions or recommendations for improving chatbot reliability would be an inappropriate concluding remark. So, we choose not to add such info.**

• In lines 50 – 58, chatbot variability was mentioned but it does not explicitly discuss potential biases in AI-generated responses. It is important to address that.

**An excellent point that we have now addressed on line 49.**

• Details on how prompts were standardized across chatbots (lines 72-74) would improve replicability.

**This information is already provided on lines 116-129.**

• Potential biases in chatbot outputs (lines 75-76) should be explicitly acknowledged.

**In line with your earlier comment, we added some text to line 49 in the introduction and further text in the limitations section of the discussion (line 650-654).**

• Recommendations for improving chatbot-generated sports nutrition advice (lines 108 - 110) should be included.

**While chatbot manufacturers should optimise their large language models by training them on the most recent sports nutrition guidelines and position statements, this study was not designed to make recommendations for improving chatbot-generated sports nutrition advice. Instead, the study was designed to compare the accuracy of nutritional knowledge between different chatbots. Therefore, adding explicit recommendations is inappropriate, and we refrain from doing so. That said, one aspect of our study was to determine how chatbot-generated outputs could be improved by using a detailed prompt containing best practice prompting techniques, including a context, a role, a specific chain of thought task, and notes to prevent lost-in-the-middle effects (e.g., Table 1 and line 98-100).**

2. Study Design (Lines 80 – 83)

• It would be beneficial to explicitly mention any potential biases introduced by modifications to the original protocol. Clearly state what amendments were made post-registration and their potential impact on findings.

**Lines 337-341 describe the amendments made post-registration. In brief, the amendments meant that Microsoft Copilot was never used in the study. This did not introduce any bias and does not impact the findings further than preventing the understanding of how Copilot performs on such tasks. We added some text to lines 647-648 to clarify the point that more chatbots should be studied in future.**

3. Table 2 - Chatbots Used (Line 129)

• The subscription-based nature of advanced models could influence accessibility and should be discussed as a potential limitation.

**We have added this info to the discussion on lines 653-654.**

4. Assumptions for Likert Scale Data (Lines 264 – 273)

• The justification for treating Likert scores as continuous when aggregated is common but should be explicitly supported by references or sensitivity analyses.

**We already included a reference (Sullivan *et al*) to justify this treatment (ref [69], line 273).**

• The log transformation of the Likert scores is reasonable, but additional justification is recommended to address concerns about its interpretability.

**Our apologies, but the sentence about the log transformation of Likert scores is incorrect. The actual process was to compile the lists of Likert scores as mean ± standard deviation so that the total score for accuracy was out of 45 and 55 for the *sports nutrition for training* and *sports nutrition for racing* domains, respectively. We have removed the sentence.**

5. Interpretation of Accuracy Categories (Lines 305 – 327)

• The categorical thresholds for percentage accuracy scores are well defined but it is unclear if these thresholds were pre-specified or data-driven, which could introduce bias.

**As described on lines 282-287, percentage accuracy scores were calculated and interpreted in line with the original Likert scale. Therefore, the categorical thresholds for percentage accuracy were pre-specified (in the manuscript and the registered protocol) because Likert scores of 1, 2, 3, 4, 5 are equivalent to percentages of <20%, >20% to ≤40%, >40% to ≤60%, >60% to ≤80%, and >80%, respectively.**

6. Clarity and Readability (lines 529 – 539)

• Some statistical results are presented in a dense manner that may be difficult for a general audience to interpret.

**Because the R-values and 95% confidence intervals are presented in the tables, we have now removed these data from the sentences on lines 529-540 to make them less dense.**

• Consider summarizing key findings in a more reader-friendly format (e.g., adding bullet points).

**Thank you for the suggestion to summarise key findings in a bullet-point format. While we understand that your intent is to enhance readability, we have chosen to retain the current narrative structure that fits within the guidelines of PLOS ONE (for which bullet point summary findings are not a formal requirement). We believe the narrative format better preserves the logical flow and nuanced interpretation of the findings. Additionally, we have made efforts throughout the discussion and conclusion to clearly highlight the most important takeaways for the reader.**

7. Use of Figures and Tables

• Table 5 includes multiple p-values; consider highlighting significant results for readability (Line 396).

**Table 5 (line 397) already contains the P-values in ascending order and uses *, **, and *** to indicate significance at 5%, 1%, and 0.1% levels.**

8. Experiment 2 Model Justification

• The logistic mixed model was chosen based on AIC/BIC (Lines 506 – 509), but additional justification on model assumptions (e.g., normality of residuals) would be beneficial.

**Thank you for identifying model assumptions as a point to clarify. We confirmed these assumptions by (i) verifying the** **uniformity of residuals** **via visual inspection of QQ plots and histograms, and (ii) using residual diagnostics tests for residual uniformity, residual dispersion, and** **zero inflation. All such tests revealed that the binomial model met the assumptions: residuals were uniformly distributed, contained the correct amount of variation (i.e., there was no overdispersion), and the model did not predict too many or too few zeros. We have now clarified this on lines 518-521.**

• The random intercept for ChatbotID was reported as zero (Line 509 - 510), but more details on its implications should be provided.

**Thank you for highlighting the random intercept estimate for ChatbotID. The zero random intercept for ChatbotID** **indicates that there was negligible variation in baseline performance among the individual chatbots beyond what was already captured by the fixed effects included in the model. In practical terms, this indicates that** **chatbot-specific differences in overall accuracy were effectively explained by their group membership (fixed effects) without requiring additional modelling of chatbot-level random variability. This supports our selection of a simpler model structure (random intercept-only without random slopes) and that the observed effects are generalizable across individual chatbots, rather than driven by a few outliers or idiosyncratic models. We have added some text to lines 523**-**527 to clarify this point.**

9. Grammar and Style Adjustments

• Consider rewording the quoted sentence for clarity: “The comparisons had moderate effect sizes but only low to moderate statistical power" (Line 370 – 371).

**We have reworded to “*The comparisons showed moderate effect sizes, but statistical power to detect significant differences was only low to moderate.*” (line 371-372).**

• Consider changing "are" to "is" in the quoted excerpt: "Full post hoc data for the ChatbotID × PromptType interaction are available..." (Lines 380 - 381).

**Done (line 382).**

10. Clarity and Justification of Key Findings

• The distinction between "accuracy," "completeness," and "clarity" should be defined earlier to prevent reader confusion.

**We have now provided this information on lines 62-64 in the introduction.**

• Some comparative claims about chatbot performance lack direct references to quantitative results (e.g., Lines 552 – 553: “ChatGPT4o had more complete answers”).

**Where relevant, we have now added references to the Figures and Tables in the first two paragraphs of the Discussion on lines 566-569, 572-573, 577, and 581.**

• The explanation for why chatbots struggled with certain topics (e.g., hydration, energy availability) should be expanded with references to chatbot training limitations.

**We have now addressed this point with some additional text about training data bias and recency bias on lines 628 and 650-654.**

11. Statistical Power and Methodological Transparency

• Experiment 1 is noted as underpowered (Lines 661 – 662). The authors should provide details on the required sample size to detect meaningful differences.

**Excellent point. We found 6 statistically significant differences among various chatbots (see Table 5, line 397) with Cohen’s D effect sizes ranging 0.502 to 0.796 and statistical power ranging 0.340 to 0.689. Therefore, on lines 371-372, we wrote: “***The comparisons showed moderate effect sizes, but statistical power to detect significant differences was only low to moderate***”. Using the statistically significant comparison with the smallest effect size (d=0.502) and lowest power (0.340), we recalculated the necessary sample size to detect a difference with at least 80% power, finding that 210 observations would be necessary. Therefore, with the same 6 chatbots, we would require 210/6 = 35 criteria/expected answers to obtain sufficient power, rather than the 20 criteria/expected answers we originally used. We have added some text to lines 684-686 to clarify this point.**

12. Implications for AI Development

• While the manuscript highlights the need for chatbot fine-tuning, it does not offer specific recommendations for AI developers. Adding this would enhance the practical impact of the study.

**We appreciate the reviewer’s interest in enhancing the practical implications of the study by suggesting recommendations for AI developers. However, as noted in our earlier response, the study was not designed to evaluate or engineer specific strategies for improving chatbot-generated sports nutrition advice. Rather, we aimed to benchmark the current state of knowledge accuracy across widely used generative AI chatbots. Given this comparative framework, offering prescriptive guidance to AI developers would go beyond the scope of our data and risk overinterpretation. That said, we do believe the study has important practical implications: as noted in the final sentence of the manuscript, “until the LLMs underpinning these generative AI chatbots are improved, an athlete or coach seeking tailored sports nutrition advice should seek professional input from a registered nutritionist/dietician.”. The wording in the conclusion was carefully chosen to ensure the message is clear and actionable for end-users of these technologies and to avoid overinterpretation of the findings.**
