# Supplementary material for: The sports nutrition knowledge of large language model (LLM) artificial intelligence (AI) chatbots: An assessment of accuracy, completeness, clarity, quality of evidence, and test-retest reliability
Source: PLoS One. 2025 Jun 13;20(6):e0325982. doi: 10.1371/journal.pone.0325982 (PMC12165421; doi:10.1371/journal.pone.0325982)
Supplement: Attachment — Submitted filename: Response to Reviewers.docx [file pone.0325982.s006.docx]

# Response to Reviewers

## Reviewers' comments:

### **Reviewer 1**

**Thank you for your comments. We have addressed them point-by-point in bold blue font below.**

1. The authors attribute the chatbots’ poor performance in certain areas (e.g., hydration, energy availability) mainly to general causes such as “training data bias.” However, this explanation should be expanded with more concrete examples and supported by references from the literature.

Furthermore, the manuscript would benefit from including practical suggestions for developers of AI systems. For example, it could recommend fine-tuning based on official sports nutrition guidelines. Currently, the recommendation to consult a dietitian is confined to a single sentence in the conclusion and does not sufficiently address the broader developmental implications.

**We thank Reviewer #1 for highlighting the need for greater specificity and practical implications regarding chatbot limitations. To address this, we expanded our explanation beyond general terms like “training data bias” and have added some text to lines 600-602.**

2. While the distinctions between “simple” and “detailed” prompts are well defined, the manuscript does not adequately explain why some chatbots (e.g., Claude) responded more effectively to detailed prompts. A more in-depth discussion of how different models process prompt complexity would improve the interpretation of results.

**Thank you for this comment. We already discussed this point on lines 622-624 but have included some additional detail (lines 624-630) to further explain the differences in chatbot performance based on prompt type.**

3. Although the exclusion of variables such as gender and ethnicity from the prompts is methodologically explained, the possible consequences of this decision are not addressed in the discussion. The authors should discuss how the lack of gender- or culture-specific prompts might have influenced the outputs, especially considering that nutritional needs can vary across these dimensions.

**Thank you for this comment and insight.**

**Including demographic information such as gender, ethnicity, or cultural background in our prompts *might* have elicited more tailored and potentially more effective sports nutrition guidance. However, because the majority of sports nutrition research has been conducted on a relatively homogeneous population (often young white males), chatbot responses would likely have remained generalized or simply reflected the prevailing norms of that population (**[**Turner et al., 2022**](https://pubmed.ncbi.nlm.nih.gov/35267928/)**;** [**Cowley et al., 2022**](https://pubmed.ncbi.nlm.nih.gov/35168200/)**). Furthermore, as we already mentioned on lines 109-113, current position stands and consensus statements do not provide sports nutrition guidelines specific to race, ethnicity, culture, or gender. As a result, including demographic cues may not meaningfully improve personalization and could, in fact, have inadvertently reinforced existing biases. This concern is supported by recent evidence showing that large language models can generate biased clinical recommendations when demographic information is included in prompts (Sanghavi et al., 2024** [**https://www.nature.com/articles/s41591-025-03626-6#Sec9**](https://www.nature.com/articles/s41591-025-03626-6#Sec9)**). Although this is an important issue, it is a general topic related to AI and sports nutrition ethics and is beyond the scope of the aim of our study. Therefore, we choose not to add any further text to the manuscript.**
